# Supplementary material for: Long-term nitrogen burial exceeds denitrification in global fjords
Source: Nat Commun. 2026 Apr 1;17:3148. doi: 10.1038/s41467-026-71116-5 (PMC13043757; doi:10.1038/s41467-026-71116-5)
Supplement: Supplementary file 1 — Supplementary Information [file 41467_2026_71116_MOESM1_ESM.pdf]

Supplementary Materials for

Long-term nitrogen burial exceeds denitrification in global fjords

Henry L.S. Cheung[1*], Lubrina S. Levin[2], Craig Smeaton[3], Tobia Politi[1], Bo Thamdrup[2], Isaac R. Santos[1], Stefano Bonaglia[1]

[*]Corresponding author: lok.shan.cheung@gu.se

The document includes:

- Methods.
- Figure S1 Relationship between fjord N loss processes rate and environmental factors.
- Figure S2 Spatial distributions of nitrogen accumulation rates (N_{AR}).
- Figure S3 Sediment nitrate reduction rates measured in the centre of Gullmar Fjord during oxic and hypoxic season.
- Figure S4 Rates of nitrogen cycling processes in global fjords.
- Figure S5 Potential changes in fjord N_2 production as a function of oxygen concentrations.
- Figure S6 Location of sampling sites in Sweden and Iceland.
- Figure S7 Result of spatial weighting of each measurement in the database.
- Figure S8 Summary of empirical observations and spatial weighting bootstrapped means.
- Table S1 Summary of global fjord nitrogen burial estimates.
- Table S2 Summary of the Bayesian segmented Gaussian regression model describing the relationship between N_2 production and O_2 concentration.
- Table S3 Summary of sampling site in Sweden and Iceland.
- Supplementary References.

Methods

Studied sites

New datasets of fjord nitrogen (N) loss via sediment burial and microbial N_2 production were collected in five fjords in Sweden and Iceland (Fig. S5). Gullmar, Hake, and By Fjords are temperate systems (57–59˚N) located at west coast of Sweden, with length of 28, 22, and 4 km, respectively. Bottom water of By, Gullmar, and Hake Fjords are long-term anoxic, seasonally hypoxic, and oxic conditions, respectively. Bottom waters of Gullmar Fjord are renewed annually, yet low oxygen (O_2) concentrations can be developed when deep-water exchanges is absent or only partially occur [1]. Water column below sill depth (~20 m) in By Fjord is characterised by long-term euxinic condition. The water column is mixed vertically every 3–5 years, while O_2 depleted within weeks to months by significant biological consumptions [2]. Gullmar Fjord has been designated as a conservation area with temperate forests, where minimal anthropogenic inputs have been influencing the fjord since past decades [3]. In contrast, catchment of By and Hake Fjord are covered by agriculture and developed areas. Reyðarfjörður and Berufjörður, are sub-polar systems located at the east coast of Iceland (64–65˚N). These fjords are 20–30 km long with 60–170 m water depth. The relatively small drainage basins (<160 km^2) resulted in limited river inputs to both fjords. Vegetation cover is sparse (25–45%) and is dominated by shrubs, grasses, and mosses [4]. Aquaculture has been established in both fjords, where higher organic matter contents and labilities were found in waters adjacent to sea cages [5]. Water columns were well-mixed and oxygenated in these fjords during our sampling, while hypoxic conditions (O_2 concentration < 60 μM) have been recorded near the head of Berufjörður in summer [6].

Sampling procedures

In Swedish fjords, samples for mass accumulation rates and sediment nitrogen analyses were collected from nine locations within the three fjords in September–October 2021. Measured rates and details of the sampling were described in previous publication [7]. To determine N_2 production in the three Swedish fjords, incubation experiments were conducted during sampling campaigns in 2023 and 2024. Prior to sediment sampling, CTD casts with a Sea-Bird SBE 911 were made to record bottom water column temperature, dissolved O_2, and salinity. Approximately 70 L of bottom waters were also collected for later incubation experiments. In Gullmar and Hake Fjord, sediments were collected from the seafloor using a box corer. Intact cores (inner diameter = 4.6 cm; length = 30 cm) were sub-sampled from undisturbed sediment in the corer, and were immediately submerged *in situ* bottom water at ambient temperature for later incubation experiments. In By

Fjord, N_2 production rates were determine using water samples at oxic-anoxic interface at the centre station were collected in 2023 (see details below), given that bottom water was anoxic and highly sulfidic [8]. For N_2 production at the head and mouth in By Fjord, benthic rates were obtained from a previous publication [9].

For Icelandic fjords, samples for mass accumulation rates, sediment stoichiometric analysis, and N_2 production measurements were collected in June 2023. Sediment cores and bottom waters were collected by the same sampling approached as in Swedish fjords. Larger intact cores (inner diameter = 5.6 cm; length = 44 cm) and smaller ones (inner diameter = 4.6 cm; length = 30 cm) were sub-sampled from the box corer for sediment analyses and incubation experiments, respectively. Larger cores were sliced at 1 cm intervals from 0-6 cm, 2 cm intervals from 6-20 cm, then a 5 cm interval from 20 – 25 cm immediately after sampling. Samples were stored in sealed LDPE containers at -20°C for later sediment analyses. Mass accumulation rates were determined based on particle-reactive radiotracer-derived age models in the sediment core. Sediments for total nitrogen (TN%) were weighed into tin capsules with no acid treatment, and analysed on a Thermo Electron DeltaV Advantage isotope ratio mass spectrometer coupled with a ConFlo II interface linked to a Carlo Erba NA 1500 CNHS Elemental Analyzer. Smaller cores were immediately submerged *in situ* bottom water at ambient temperature for later incubation experiments.

<u>Sediment N_2 production processes rate measurement and calculation</u>

Sediment N_2 production rates (denitrification and anammox) as well as dissimilatory nitrate reduction to ammonium rates (DNRA) were determined by intact sediment incubation using revised-isotope pairing technique [10]. Collected sediment cores in each station of Swedish and Icelandic fjords were transferred to incubation tanks situated in a temperature-controlled room at *in situ* bottom water temperature (Table S4). *In situ* bottom water in each incubation tanks were air-aerated (oxic stations) and well- stirred. For hypoxic stations (i.e., Gullmar Fjord in October, 2024), O_2 concentrations of the water in the incubation tanks were maintained at *in situ* concentration by aerating N_2/Air mixture that adjusted with flowmeters. O_2 concentrations in the tanks were monitored by an optode probe (OXROB10, PyroScience). A temperature sensor (TDIP15, PyroScience) was placed into a water bath for real-time temperature compensation of optode measurements. Each core was supplied with a magnetic stirring bar (60 rpm) to maintain the water phase of each core well mixed.

After an overnight preincubation, sediment denitrification, anammox, and DNRA rates were measured by ^{15}N-nitrate ($^{15}NO_3^-$) amendments. Briefly, sediment cores (n = 12) were spiked with $^{15}NO_3^-$ tracer to obtain triplicate cores with mean final concentrations of 11, 24, 56, and 120 μM ^{15}N. To calculate actual ^{15}N enrichment in each core, water samples for the NO_3^- concentration analysis were collected before and after the tracer addition, and analysed by spectrophotometric method [11]. Incubation started after a lag time of 1- 4 hrs to allow the spiked $^{15}NO_3^-$ to homogeneously mix with the endogenous $^{14}NO_3^-$ and diffused to the sediment nitrate reduction zone. Each core was then capped with airtight lids, and O_2 concentrations were recorded immediately via optode spots (OXSP5, PyroScience). Incubations were terminated by uncapping the core when O_2 had decreased by 20 – 25% of the initial concentrations, in order to ensure linear uptake of O_2 and solute exchange over incubation time (oxic: 12 – 24 hrs; hypoxic: 6 hrs). Top 6 cm of sediment of each core were gently mixed with overlying water to obtain a slurry. A 20 mL slurry aliquot was immediately transferred into a 12 mL exetainer (Labco) and fixed with 200 μL of 50% w/v $ZnCl_2$ for later $^{29}N_2$ and $^{30}N_2$ analyses. To determine the rate of DNRA, a sub-sample was collected from the slurry samples for $^{29}N_2$ and $^{30}N_2$ analyses for later $^{15}NH_4^+$ analysis. Abundance of $^{29}N_2$ and $^{30}N_2$ of N_2 is determined directly through headspace analysis by gas chromatography-isotope ratio mass spectrometry (GC-IRMS), while $^{15}NH_4^+$ can be first treated with hypobromite-iodine solution that oxidize NH_4^+ to N_2 form, and the result content of $^{29}N_2$ and $^{30}N_2$ measured in GC-IRMS represent which originally from the $^{15}NH_4^+$ in the sample.

Total N_2 production (p_{14}) was expressed as a function of r_{14}, the ratio between $^{14}NO_3^-$ and $^{15}NO_3^-$ in the sediment nitrate reduction zone:

$$p_{14} = 2 \times r_{14}\left(p^{29}N_2 + p^{30}N_2(1 - r_{14})\right) \quad (1)$$

where $p^{29}N_2$ and $p^{30}N_2$ are the production rate of $^{29}N_2$ and $^{30}N_2$ from the incubation, which are calculated as the slopes of the significant ($p < 0.05$) linear regression of $^{29}N_2$ and $^{30}N_2$ concentration as a function of time. The value of r_{14} is calculated from the $p^{29}N_2$ and $p^{30}N_2$ measured in two incubations with different $^{15}NO_3^-$ concentrations:

$$r_{14}{}^{(a)} = \frac{p^{29}N_2{}^{(a)} - V \times p^{29}N_2{}^{(b)}}{2 \times (p^{30}N_2{}^{(a)} - V^2 \times p^{30}N_2{}^{(b)})} \quad (2)$$

where $p^{29}N_2^{(a)}$ and $p^{30}N_2^{(a)}$ refer to the production rates of $^{29}N_2$ and $^{30}N_2$ in the incubation at lower $^{15}NO_3^-$ concentration, whereas $p^{29}N_2^{(b)}$ and $p^{30}N_2^{(b)}$ refer to the production rates of $^{29}N_2$ and $^{30}N_2$ in the incubation at higher $^{15}NO_3^-$ concentration. The parameter V, the ratio between concentrations of $^{15}NO_3^-$ in the water phase from two incubations with different $^{15}NO_3^-$ concentrations, is calculated by:

$$V = \frac{p^{29}N_2{}^{(a)} + 2 \times p^{30}N_2{}^{(a)}}{p^{29}N_2{}^{(b)} + 2 \times p^{30}N_2{}^{(b)}} \quad (3)$$

Values of V and r_{14} are calculated by matrix of values from 4 different $^{15}NO_3^-$ amendments using Eqs. 2 and 3.

Anammox rate (A_{14}) was calculated according to the formula:

$$A_{14} = 2 \times r_{14}(p^{29}N_2 - 2 \times r_{14} \times p^{30}N_2) \quad (4)$$

while the ambient denitrification rate (D_{14}) is calculated by subtracting the anammox rate from p_{14}:

$$D_{14} = p_{14} - aao_{14} \quad (5)$$

Rates of DNRA are calculated by multiplying the production rate of $^{15}NH_4^+$ ($p^{15}NH_4^+$, i.e., the increase in $^{15}NH_4^+$ concentrations versus time) by r_{14}.

<u>Water column N_2 production rate measurement</u>

Rates of water column total N_2 production were determined using ^{15}N-labelled substrates. Experiments were conducted with water sampled near the oxic-anoxic interface (21, 22.5 and 24 m below surface) at the centre station of By Fjord following the procedures described previously [12, 13]. For a rate profile, a 2 L screw top bottle was filled directly from the Niskin bottle at each of three depths, overflowed at least three times the total volume, and immediately sealed bubble-free with a butyl rubber stopper. Collected samples were stored for 6 hours in the dark at *in situ* temperature (8 °C) until processed. To set up the experiment, water was removed from the bottle to allow He purging for 45 min. After purging, 1.6 mL of 100 mM He-purged NaS was added to the bottle to mimic the ambient sulphidic condition (38 μM final concentration) and transferred into 12 mL exetainers (LabCo) and capped with He-degassed caps, and headspaced with He to minimise O_2 contamination [14, 15]. To determine potential denitrification rates as accumulation of $^{30}N_2$, samples received a 2 mL He headspace and amended to a finial concentration of 20.0 μM $^{15}NO_3^-$ in duplicate samples per time point (0, 3, 6, 9, and 12 hrs). To determine N loss kinetics, samples received a 2mL He headspace and amended to a finial concentration of 0.3, 0.7, 1.4, 3.6, 8.1, and 11.0 μM $^{15}NO_3^-$ in duplicate samples per time point (0.5, 2, 5, and 10 hrs). and left to incubate in the dark at *in situ* temperature. At each time point, incubation was terminated by adding 100 µL of 50% w/v $ZnCl_2$ solution to each exetainer. Final total NO_3^- concentrations were analysed on an ion chromatograph on samples from the first time point of each treatment.

Water column microbial N loss rates were quantified from the combined production of ^{15}N labelled N_2 and N_2O as determined by GC-IRMS (Dalsgaard et al. 2012). With the lower $^{15}NO_3^-$

amendments used for determination of N loss kinetics, the NO_3^- pool was depleted by ≥ 50% during the incubation, and rates of N_2 + N_2O production decreased over time due to increasing substrate limitation. As limitation may even have affected the first time interval of the incubation at the lowest amendments, leading to an underestimation of initial rates, kinetic parameters were determined by fitting the incubation time, *t*, by which N_2 + N_2O had accumulated to a certain observed concentration, N_{loss}, to the time by which this N loss would occur assuming that the depletion of the substrate (NO_3^-) from an initial concentration, N_{init}, was subject to Michaelis-Menten kinetics [16]:

$$t = \left[N_{loss} + K_m \times \ln\left(\frac{N_{init}}{N_{init} - N_{loss}}\right)\right] \times {V_{max}}^{-1} \quad (6)$$

where K_m and V_{max} are the parameters of the Michaelis-Menten equation:

$$Rate = \frac{V_{max} \times [{NO_3}^-]}{K_m + [{NO_3}^-]} \quad (7)$$

Thus, K_m and V_{max} were estimated by a single least-squares fit of Equation 6 across the whole range of $^{15}NO_3^-$ amendments, yielding values of 0.58 μM h^{-1} and 0.33 μM, respectively. Ambient microbial N loss rates were estimated using the Michaelis-Menten equation (7) with derived values K_m and *in situ* NO_3^- concentrations, where potential denitrification rates were applied as V_{max} [17]. The resulting volumetric rates were then converted into areal rates by multiplying with the depth of the denitrification zone, which was defined as the thickness of the anoxic NO_3^- containing layers at the oxic-anoxic interface [13]. The depth was identified when the O_2 sensor was pulled slowly upwards from the anoxic bottom waters to the oxygenated surface waters. Before the upcast, the O_2 sensor stayed at the anoxic bottom water for a period of 5 min to obtain signal for zero O_2. Water column profiles of NO_3^- concentrations were measured on 6 discrete samples from the Niskin bottles at 1 m intervals near the oxic-anoxic interface. NO_3^- concentrations were analysed as described above.

Supplementary Figures

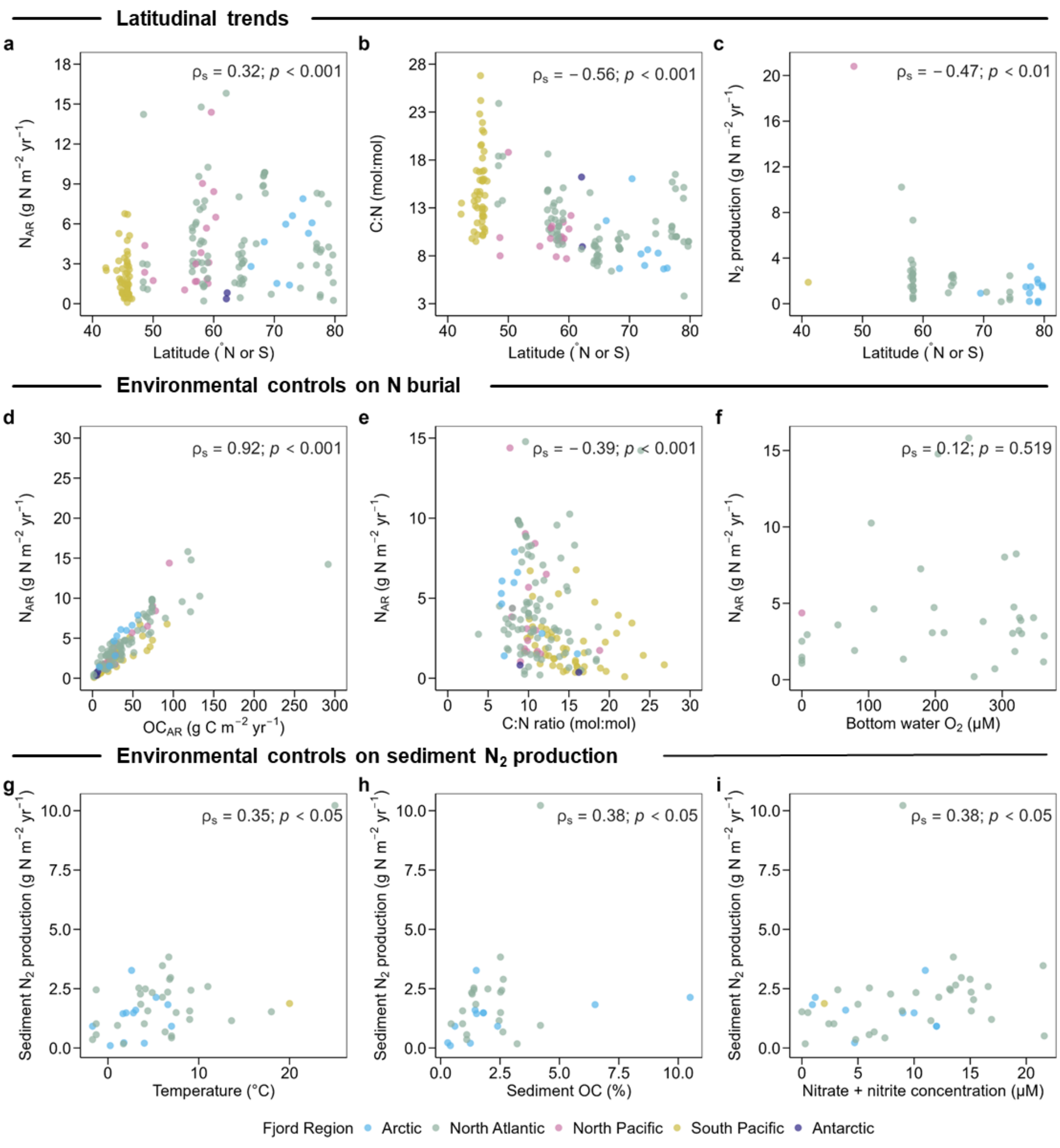

Figure S1 Relationship between fjord N loss process rate and environmental factors. (**a-c**) Latitudinal trends in fjord nitrogen burial (N_{AR}), sediment C:N ratio, and fjord N_2 production across fjord sites, respectively. **(d-f)** Environmental controls on fjord N_{AR}, including organic carbon accumulation rates (OC_{AR}), sediment C:N ratio, and bottom water oxygen (O_2) concentration, respectively. **(g-i)** Environmental controls on fjord sediment N_2 production rate (water column production excluded), including bottom water temperature, sediment organic carbon content (OC), and bottom water nitrate + nitrite concentration, respectively. Data points are colour-coded on the basis of fjord regions. Spearman correlation coefficient (ρ_s) and corresponding p-value are shown.

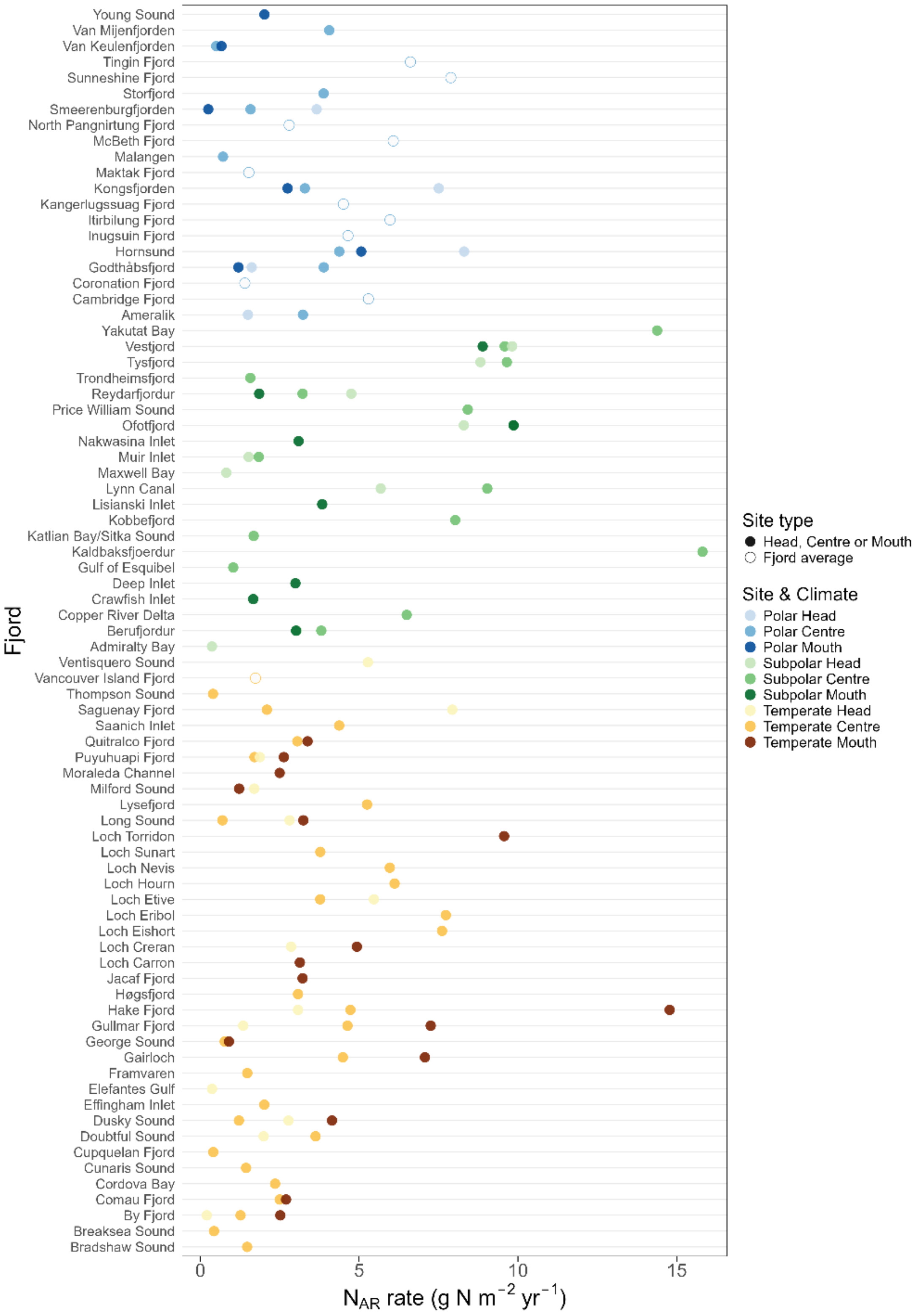

Figure S2 Spatial distributions of nitrogen accumulation rates (N_{AR}). Rates were determined from cores collected at the head, centre, and mouth of the corresponding fjords across climate regions (colour-coded). Solid dots indicate rates at head, centre, and mouth of the fjord, whilst circles indicate fjord average rates.

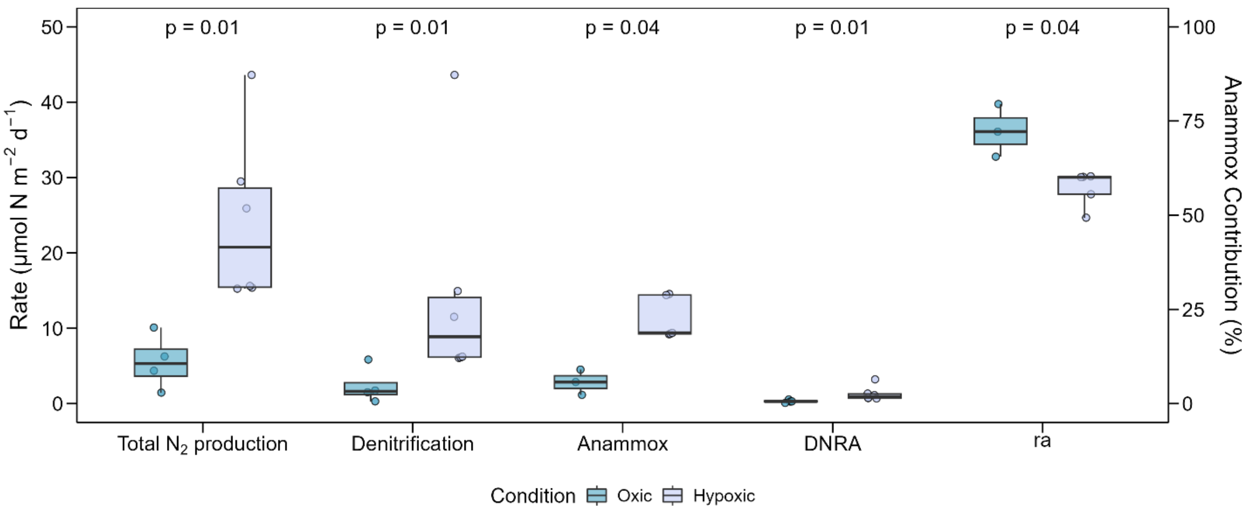

Figure S3 Sediment nitrate reduction rates measured in the centre of Gullmar Fjord during oxic and hypoxic season. Mean N_2 production rates (denitrification and anammox) and dissimilatory nitrate reduction to ammonium rates (DNRA) were measured by sediment core ^{15}N-labelled experiments. Sediments were collected during oxic (April 2023) and hypoxic (October 2024) conditions that oxygen concentrations were 260 and 61 μM, respectively. Anammox contribution to total N_2 production (ra) were defined as the anammox rates relative to the sum of denitrification and anammox rates. Error bars denote standard errors. Denoted *p*-values were resulted from Mann-Whitney-Wilcoxon test between oxic and hypoxic rates of the corresponding process.

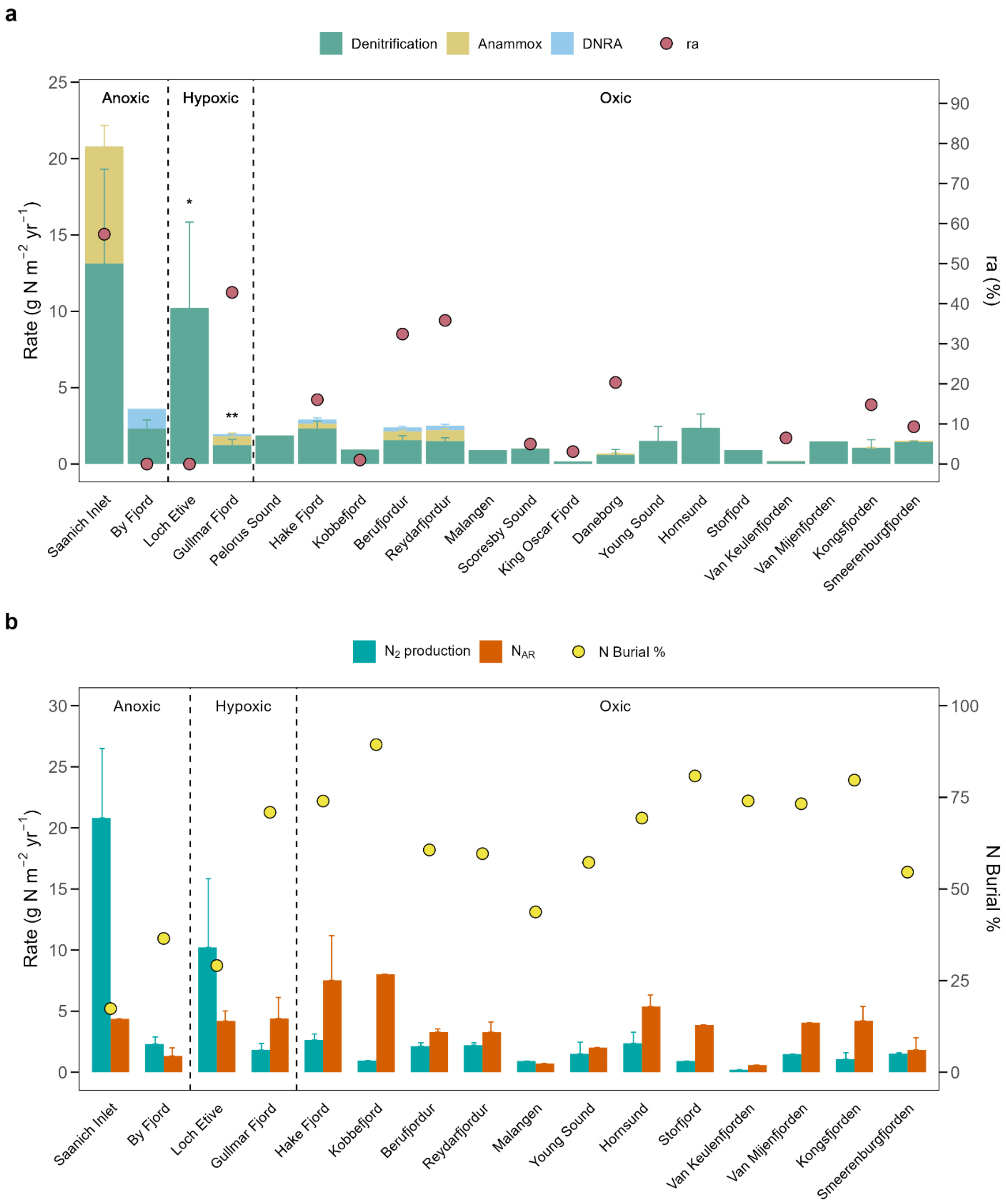

Figure S4 Rates of nitrogen cycling processes in global fjords. (**a**) Mean total N_2 production rates (denitrification and anammox) and dissimilatory nitrate reduction to ammonium rates (DNRA) measured by ^{15}N-labelled experiments. Anammox contribution to total N_2 production (ra) were defined as the anammox rates relative to the total microbial N_2 production rate (denitrification + anammox). Rates in oxic and hypoxic fjords were determined from the sediments, while rates in anoxic fjords were determined from the nitrate-reduction zone along the water column. * denotes N_2 production rate measured under oxic condition in seasonal hypoxic Loch Etive, and ** denotes rates measured under both oxic and hypoxic condition in Gullmar Fjord. (**b**) Mean total N_2 production rate and sediment nitrogen accumulation rate (N_{AR}) in fjords where both processes were measured. Dots denote relative contribution of sediment to total N loss (N_2 production + N_{AR}). Vertical line divided fjords with anoxic ($O_2 < 10$ μM), hypoxic (O_2 10 – 100 μM), and oxic ($O_2 > 100$ μM) fjords. Fjords are ranks with increasing latitude (from left to right) within each redox condition group. Error bars denote standard errors.

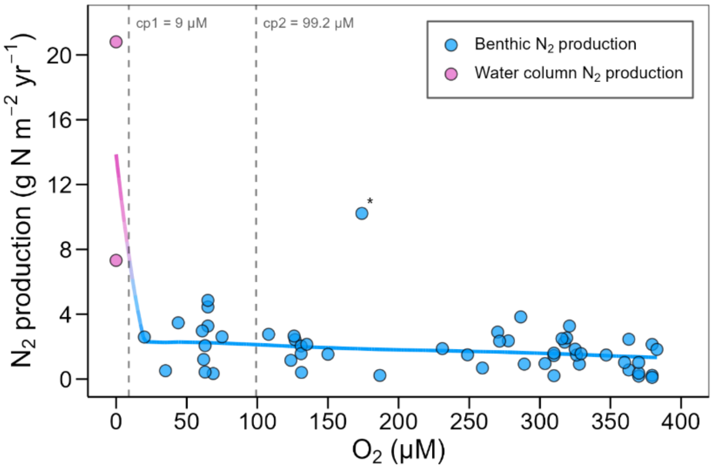

Figure S5 Potential changes in fjord N_2 production as a function of oxygen concentrations. Sediment and water column total N_2 production rates (denitrification + anammox) and N burial rates in global fjords are based on a compilation of measurements from the literature and new data from Sweden and Iceland. A Bayesian segmented regression model was fitted to the observed N_2 production rates. Dashed lines indicate the estimated change points (cp1 and cp2) of shifts in N_2 production rate. An asterisk denotes a higher benthic N_2 production rate reported in Loch Etive under oxygenated conditions, yet this system is known to experience periodic hypoxia [18].

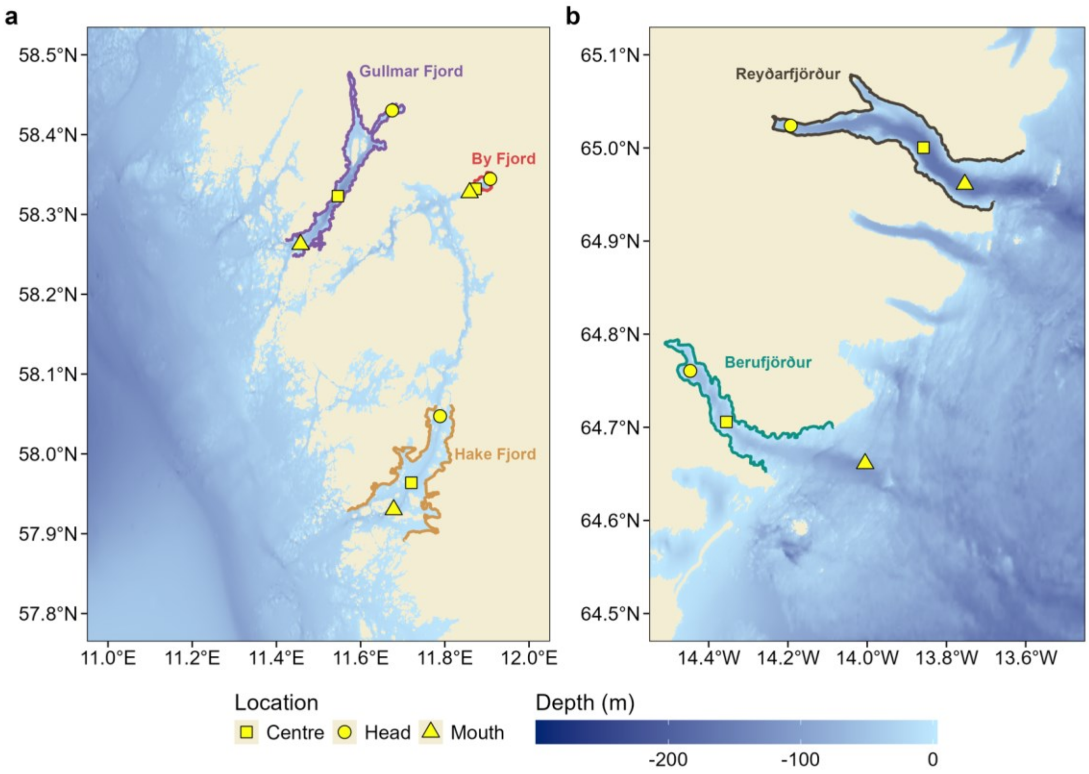

Figure S6 Location of sampling sites in Sweden and Iceland. Samples for fjord nitrogen burial and microbial N_2 production measurements were collected from five fjords in **(a)** Sweden and **(b)** Iceland during sampling campaigns in 2023 and 2024. Bolded, colour-coded coastlines indicate fjord sampled (purple: Gullmar Fjord; red: By Fjord; orange: Hake Fjord; brown: Reyðarfjörður; green: Berufjörður). Symbol shapes indicate each sampling site within each fjord (square: centre; circle: head; triangle: mouth). The blue colour gradient represents water depth.

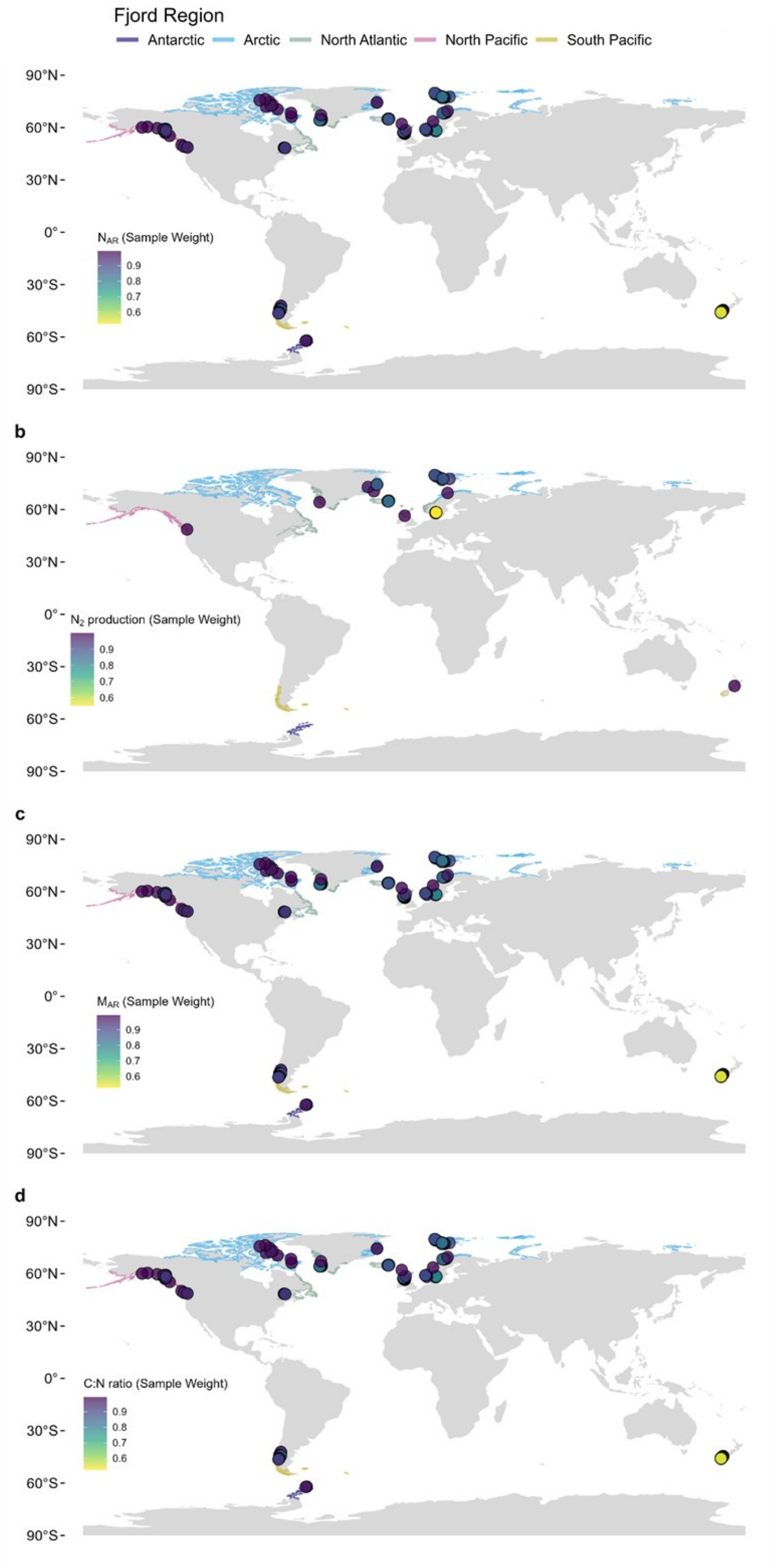

Figure S7 Result of spatial weighting of each measurement in the database. Each observation of (**a**) nitrogen accumulation rate (N_{AR}), (**b**) N_2 production rate, **(c)** mass accumulation rate (M_{AR}), and **(d)** sediment

C:N ratio is assigned with weights based on their spatial proximity to other samples with spatial scale of approximately 55 km (0.5 degree). Bolded, colour-coded coastlines indicate fjord regions.

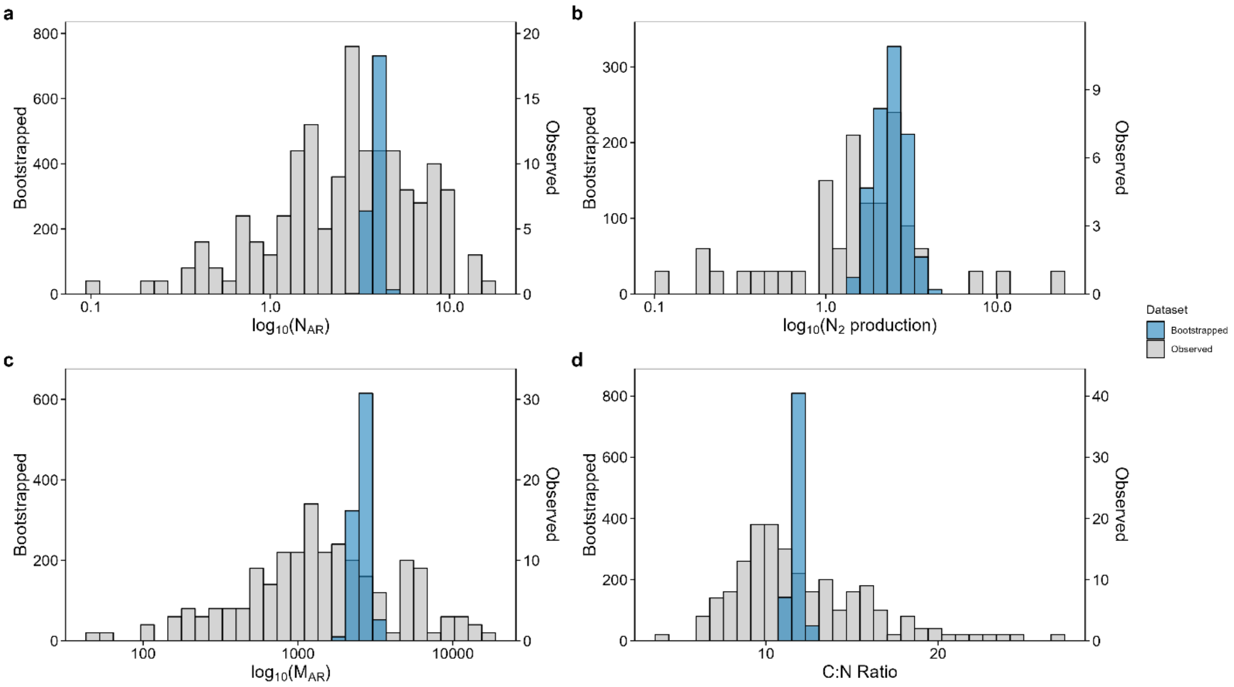

Figure S8 Summary of empirical observations and spatial weighting bootstrapped means. Bootstrap analysis was performed to the dataset of spatial weighted **(a)** nitrogen accumulation rate (N_{AR}), **(b)** N_2 production rate (N_2), **(c)** mass accumulation rate (M_{AR}), and **(d)** sediment C:N ratio to generate distributions of 1,000 weighted bootstrapped means. Distribution of observed raw data (grey) and bootstrapped data (blue) used in the analyses were illustrated. Note that x-axes for N_{AR}, N_2 production, and M_{AR} are log-transformed.

Supplementary Table

Table S1 Summary of global fjord nitrogen burial estimates.

	**Min**	**Q1**	**Median**	**Q3**	**Max**
Marine N burial (Tg N yr^{-1}) [19]	10.0	17.0	22.5	25.0	58.0
**Fjord area 1 (259,899 km^2) [20]**					
Fjord N burial (Tg N yr^{-1})	0.8	1.0	1.0	1.1	1.2
Fjord % of total	1.7	4.0	4.5	5.9	10.1
**Fjord area 2 (455,535 km^2) [21]**					
Fjord N burial (Tg N yr^{-1})	1.4	1.7	1.8	1.9	2.1
Fjord % of total	3.0	7.1	7.8	10.4	17.6

Table S2 Summary of the Bayesian segmented Gaussian regression model describing the relationship between N_2 production and O_2 concentration.

Parameter	Mean (O_2, μM)	Lower 95% intervals	Upper 95% intervals	$\hat{R}$	Effective sample size
cp_1	9.0	0.0	18.6	1.003	18546
cp_2	99.2	0.8	324.9	1.001	11874
σ	1.7	1.4	2.1	1.000	29460

Note. cp_1 and cp_2 are change points of the segments, σ denotes the residual standard deviation, and $\hat{R}$ is the potential scale reduction factor.

Table S3 Summary of sampling site in Sweden and Iceland.

Region	Fjord	Location	Depth (m)	Redox condition	Temperature (°C)	O_2 (μM)	NH_4^+ (μM)	NO_x^- (μM)
Sweden West Coast	Gullmar Fjord	Head	26	Oxic	14.0	124	0.4	12.0
		Centre	118	Seasonally hypoxic	6.3 – 6.9	61 – 259	0.5 – 2.4	6.4 – 14.2
		Mouth	57	Seasonally hypoxic	9.1	127	0.4	13.0
	Hake Fjord	Head	21	Oxic	6.7	287	2.9	13.5
		Centre	32	Oxic	7.0	265	1.9	7.8
		Mouth	41	Oxic	6.8	270	2.5	15.0
	By Fjord	Head	11	Oxic	7.0	258	<d.l.	<d.l.
		Centre	47	Anoxic	7.9	<d.l.	<d.l.	<d.l.
		Mouth	23	Anoxic	8.3	<d.l.	14.9	3.4
Iceland East Coast	Reyðarfjörður	Head	70	Oxic	3.6	318	3.3	7.9
		Centre	160	Oxic	3.6	325	3.0	5.0
		Mouth	157	Oxic	3.4	319	2.2	6.0
	Berufjörður	Head	49	Oxic	4.1	316	4.6	13.2
		Centre	52	Oxic	4.9	272	4.2	10.2
		Mouth	114	Oxic	4.4	329	1.9	8.9

Note. Site water depth, bottom water temperature, dissolved O_2 concentration (O_2), ammonium (NH_4^+), and nitrate + nitrate (NO_x^-) are shown. Ranges of measured values are reported for centre station of Gullmar Fjord, where samples were collected from two campaigns in oxic and hypoxic seasons. <d.l. denotes below detection limits.

Supplementary References [Including global fjord dataset references which are not cited in the main text]

1. Filipsson H. A 200-year environmental record of a low-oxygen fjord, sweden, elucidated by benthic foraminifera, sediment characteristics and hydrographic data. *Journal of Foraminiferal Research - J FORAMIN RES* **34**, 277-293 (2004).

2. Viktorsson L, Kononets M, Roos P, Hall P. Recycling and burial of phosphorus in sediments of an anoxic fjord—The By Fjord, western Sweden. *Journal of Marine Research* **71**, (2013).

3. Nordberg K, Gustafsson M, Krantz A-L. Decreasing oxygen concentrations in the Gullmar Fjord, Sweden, as confirmed by benthic foraminifera, and the possible association with NAO. *Journal of Marine Systems* **23**, 303-316 (2000).

4. Denk T, Grímsson F, Zetter R, Símonarson L. *Late Cainozoic Floras of Iceland: 15 Million Years of Vegetation and Climate History in the Northern Atlantic* (2011).

5. Eiriksson T*, et al. Estimate of organic load from aquaculture – a way to increased sustainability* (2017).

6. Árnadóttir AL. *Seasonal variability of dissolved oxygen in Berufjörður* (2024).

7. Watts EG*, et al.* Burial of Organic Carbon in Swedish Fjord Sediments: Highlighting the Importance of Sediment Accumulation Rate in Relation to Fjord Redox Conditions. *Journal of Geophysical Research: Biogeosciences* **129**, e2023JG007978 (2024).

8. Deutsch B, Forster S, Wilhelm M, Dippner JW, Voss M. Denitrification in sediments as a major nitrogen sink in the Baltic Sea: an extrapolation using sediment characteristics. *Biogeosciences* **7**, 3259-3271 (2010).

9. De Brabandere L*, et al.* Oxygenation of an anoxic fjord basin strongly stimulates benthic denitrification and DNRA. *Biogeochemistry* **126**, 131-152 (2015).

10. Risgaard-Petersen N, Nielsen LP, Rysgaard S, Dalsgaard T, Meyer RL. Application of the isotope pairing technique in sediments where anammox and denitrification coexist. *Limnology and Oceanography: Methods* **1**, 63-73 (2003).

11. García-Robledo E, Corzo A, Papaspyrou S. A fast and direct spectrophotometric method for the sequential determination of nitrate and nitrite at low concentrations in small volumes. *Marine Chemistry* **162**, 30-36 (2014).

12. Bonaglia S, Klawonn I, De Brabandere L, Deutsch B, Thamdrup B, Brüchert V. Denitrification and DNRA at the Baltic Sea oxic–anoxic interface: Substrate spectrum and kinetics. *Limnology and Oceanography* **61**, 1900-1915 (2016).

13. Dalsgaard T, De Brabandere L, Hall POJ. Denitrification in the water column of the central Baltic Sea. *Geochimica et Cosmochimica Acta* **106**, 247-260 (2013).

14. De Brabandere L*, et al.* Vertical partitioning of nitrogen-loss processes across the oxic-anoxic interface of an oceanic oxygen minimum zone. *Environmental Microbiology* **16**, 3041-3054 (2014).

15. Steinsdóttir HGR, Schauberger C, Mhatre S, Thamdrup B, Bristow LA. Aerobic and anaerobic methane oxidation in a seasonally anoxic basin. *Limnology and Oceanography* **67**, 1257-1273 (2022).

16. Dalsgaard T, Bak F. Nitrate Reduction in a Sulfate-Reducing Bacterium, Desulfovibrio desulfuricans, Isolated from Rice Paddy Soil: Sulfide Inhibition, Kinetics, and Regulation. *Applied and Environmental Microbiology* **60**, 291-297 (1994).

17. Michiels CC*, et al.* Rates and Pathways of N2 Production in a Persistently Anoxic Fjord: Saanich Inlet, British Columbia. *Frontiers in Marine Science* **6**, (2019).

18. Black KD, Gontarek S, Gillibrand P. Identifying the Risk of Deoxygenation in Scottish Sea Lochs with Isolated Deep Water. *Scottish Association for…*, (2006).

19. Zhang X, Ward BB, Sigman DM. Global Nitrogen Cycle: Critical Enzymes, Organisms, and Processes for Nitrogen Budgets and Dynamics. *Chemical Reviews* **120**, 5308-5351 (2020).

20. Laruelle GG, Rosentreter JA, Regnier P. Extrapolation-Based Regionalized Re-evaluation of the Global Estuarine Surface Area. *Estuaries and Coasts* **48**, 34 (2024).

21. Dürr HH, Laruelle GG, van Kempen CM, Slomp CP, Meybeck M, Middelkoop H. Worldwide Typology of Nearshore Coastal Systems: Defining the Estuarine Filter of River Inputs to the Oceans. *Estuaries and Coasts* **34**, 441-458 (2011).

22. á Norði G, Glud RN, Simonsen K, Gaard E. Deposition and benthic mineralization of organic carbon: A seasonal study from Faroe Islands. *Journal of Marine Systems* **177**, 53-61 (2018).

23. Buydens M*, et al.* Carbon Burial in two Greenland Fjords: Exploring the Influence of Glacier Type on Organic Carbon Dynamics. *EGUsphere* **2025**, 1-29 (2025).

24. Duffield C, Alve E, Andersen N, Andersen T, Hess S, Strohmeier T. Spatial and temporal organic carbon burial along a fjord to coast transect: A case study from Western Norway. *The Holocene* **27**, 1325-1339 (2017).

25. Faust JC, Knies J. Organic Matter Sources in North Atlantic Fjord Sediments. *Geochemistry, Geophysics, Geosystems* **20**, 2872-2885 (2019).

26. Faust JC, Knies J, Slagstad T, Vogt C, Milzer G, Giraudeau J. Geochemical composition of Trondheimsfjord surface sediments: Sources and spatial variability of marine and terrigenous components. *Continental Shelf Research* **88**, 61-71 (2014).

27. Ingall E, Kolowith L, Lyons T, Hurtgen M. Sediment carbon, nitrogen and phosphorus cycling in an anoxic fjord, Effingham Inlet, British Columbia. *American Journal of Science* **305**, 240-258 (2005).

28. Knudson KP, Hendy IL, Neil HL. Re-examining Southern Hemisphere westerly wind behavior: insights from a late Holocene precipitation reconstruction using New Zealand fjord sediments. *Quaternary Science Reviews* **30**, 3124-3138 (2011).

29. Koziorowska K, Kuliński K, Pempkowiak J. Deposition, return flux, and burial rates of nitrogen and phosphorus in the sediments of two high-Arctic fjords. *Oceanologia* **60**, 431-445 (2018).

30. Mayr C*, et al.* Responses of nitrogen and carbon deposition rates in Comau Fjord (42°S, southern Chile) to natural and anthropogenic impacts during the last century. *Continental Shelf Research* **78**, 29-38 (2014).

31. McQuoid MR, Whiticar MJ, Calvert SE, Pedersen TF. A post-glacial isotope record of primary production and accumulation in the organic sediments of Saanich Inlet, ODP Leg 169S. *Marine Geology* **174**, 273-286 (2001).

32. Ramirez MT*, et al.* Modern deposition rates and patterns of organic carbon burial in Fiordland, New Zealand. *Geophysical Research Letters* **43**, 11,768-711,776 (2016).

33. Schüller SE, Bianchi TS, Li X, Allison MA, Savage C. Historical Reconstruction of Phytoplankton Composition in Estuaries of Fiordland, New Zealand: the Application of Plant Pigment Biomarkers. *Estuaries and Coasts* **38**, 56-71 (2015).

34. Schüller SE, Savage C. Spatial distribution of diatom and pigment sedimentary records in surface sediments in Doubtful Sound, Fiordland, New Zealand. *New Zealand Journal of Marine and Freshwater Research* **45**, 591-608 (2011).

35. Sepúlveda J, Pantoja S, Hughen KA. Sources and distribution of organic matter in northern Patagonia fjords, Chile (~44–47°S): A multi-tracer approach for carbon cycling assessment. *Continental Shelf Research* **31**, 315-329 (2011).

36. Skei J. Geochemical and sedimentological considerations of a permanently anoxic fjord — Framvaren, south Norway. *Sedimentary Geology* **36**, 131-145 (1983).

37. Smeaton C, Yang H, Austin WEN. Carbon burial in the mid-latitude fjords of Scotland. *Marine Geology* **441**, 106618 (2021).

38. Smittenberg RH, Hopmans EC, Schouten S, Hayes JM, Eglinton TI, Sinninghe Damsté JS. Compound-specific radiocarbon dating of the varved Holocene sedimentary record of Saanich Inlet, Canada. *Paleoceanography* **19**, (2004).

39. Sørensen HL*, et al.* Seasonal carbon cycling in a Greenlandic fjord: an integrated pelagic and benthic study. *Marine Ecology Progress Series* **539**, 1-17 (2015).

40. St-Onge G, Hillaire-Marcel C. Isotopic constraints of sedimentary inputs and organic carbon burial rates in the Saguenay Fjord, Quebec. *Marine Geology* **176**, 1-22 (2001).

41. Syvitski JPM, LeBlanc KWG, Cranston RE. The flux and preservation of organic carbon in Baffin Island fjords. *Geological Society, London, Special Publications* **53**, 177-199 (1990).

42. Thamdrup B, Fleischer S. Temperature dependence of oxygen respiration, nitrogen mineralization, and nitrification in Arctic sediments. *Aquatic Microbial Ecology* **15**, 191-199 (1998).

43. Velinsky DJ, Fogel ML. Cycling of dissolved and particulate nitrogen and carbon in the Framvaren Fjord, Norway: stable isotopic variations. *Marine Chemistry* **67**, 161-180 (1999).

44. Walinsky SE, Prahl FG, Mix AC, Finney BP, Jaeger JM, Rosen GP. Distribution and composition of organic matter in surface sediments of coastal Southeast Alaska. *Continental Shelf Research* **29**, 1565-1579 (2009).

45. Walsh EM, Ingalls AE, Keil RG. Sources and transport of terrestrial organic matter in Vancouver Island fjords and the Vancouver-Washington Margin: A multiproxy approach using d13Corg, lignin phenols, and the ether lipid BIT index. *Limnology and Oceanography* **53**, 1054-1063 (2008).

46. Wang Y, Ahad JME, Mucci AO, Gélinas Y, Douglas PMJ. Large burial flux of modern organic carbon in the St. Lawrence estuarine system indicates a substantial atmospheric carbon sink. *Earth and Planetary Science Letters* **652**, 119204 (2025).

47. Wehrmann LM*, et al.* Iron-controlled oxidative sulfur cycling recorded in the distribution and isotopic composition of sulfur species in glacially influenced fjord sediments of west Svalbard. *Chemical Geology* **466**, 678-695 (2017).

48. Wehrmann LM*, et al.* Iron and manganese speciation and cycling in glacially influenced high-latitude fjord sediments (West Spitsbergen, Svalbard): Evidence for a benthic recycling-transport mechanism. *Geochimica et Cosmochimica Acta* **141**, 628-655 (2014).

49. Yoon HI, Park BK, Kim Y, Kim D. Glaciomarine sedimentation and its paleoceanographic implications along the fjord margins in the South Shetland Islands, Antarctica during the last 6000 years. *Palaeogeography, Palaeoclimatology, Palaeoecology* **157**, 189-211 (2000).

50. Anschutz P, Sundby B, Lefrançois L, Luther GW, Mucci A. Interactions between metal oxides and species of nitrogen and iodine in bioturbated marine sediments. *Geochimica et Cosmochimica Acta* **64**, 2751-2763 (2000).

51. Christensen PB, Glud RN, Dalsgaard T, Gillespie P. Impacts of longline mussel farming on oxygen and nitrogen dynamics and biological communities of coastal sediments. *Aquaculture* **218**, 567-588 (2003).

52. Katsev S, Chaillou G, Sundby B, Mucci A. Effects of progressive oxygen depletion on sediment diagenesis and fluxes: A model for the lower St. Lawrence River Estuary. *Limnology and Oceanography* **52**, 2555-2568 (2007).

53. Politi T*, et al.* Benthic metabolism and macrofauna bioturbation along a glacier-driven gradient in Kongsfjorden. *Estuarine, Coastal and Shelf Science* **320**, 109304 (2025).

54. Wang F, Juniper SK, Pelegrē[illegible]SIP, Macko SA. Denitrification in sediments of the Laurentian Trough, St. Lawrence Estuary, Québec, Canada. *Estuarine, Coastal and Shelf Science* **57**, 515-522 (2003).
